# Preoperative Differentiation of Combined Hepatocellular-Cholangiocarcinoma From Hepatocellular Carcinoma and Intrahepatic Cholangiocarcinoma: A Nomogram Based on Ultrasonographic Features and Clinical Indicators

**DOI:** 10.3389/fonc.2022.757774

**Published:** 2022-02-15

**Authors:** Yanling Chen, Qing Lu, Weibin Zhang, Jiaying Cao, Yi Dong, Wenping Wang

**Affiliations:** ^1^ Department of Ultrasound, Zhongshan Hospital, Fudan University, Shanghai, China; ^2^ Shanghai Institute of Medical Imaging, Shanghai, China

**Keywords:** combined hepatocellular-cholangiocarcinoma, hepatocellular carcinoma, intrahepatic cholangiocarcinoma, contrast-enhanced ultrasound, nomogram

## Abstract

**Objective:**

To establish a predictive nomogram to distinguish combined hepatocellular-cholangiocarcinoma (CHC) from hepatocellular carcinoma (HCC) and intrahepatic cholangiocarcinoma (ICC) based on preoperative clinical and ultrasound findings.

**Methods:**

A total of 261 patients with pathologically confirmed primary liver cancers (PLCs) were enrolled in this retrospective study, comprising 87 CHCs, 87 HCCs, and 87 ICCs matched by propensity score matching. Patients were randomly assigned to a training cohort and a validation one at the ratio of 7:3. A nomogram integrating ultrasound imaging characteristics and clinical features was established based on the independent risk factors selected by least absolute shrinkage and selection operator (LASSO) regression. The performance of the nomogram was evaluated in the training and validation cohorts in terms of discrimination, calibration, and clinical usefulness.

**Results:**

The nomogram, consisting of ultrasound imaging features (shape and margin on B-mode ultrasound, enhanced pattern on contrast-enhanced ultrasound) and clinical information [elevated alpha fetoprotein (AFP) level and serum protein electrophoresis (SPE) α1 level], showed promising performance in differentiating CHC from HCC and ICC, with the concordance index (C-index) of 0.8275 and 0.8530 in the training cohort and the validation cohort, respectively. Hosmer–Lemeshow test and the calibration curves suggested good consistency between predictions and observations. High clinical practicability was confirmed by the decision curve analysis.

**Conclusions:**

The nomogram based on clinical and ultrasound imaging characteristics showed good performance in the discrimination of CHC from other subtypes of PLC and would be valuable in clinical decision-making.

## Introduction

Combined hepatocellular-cholangiocarcinoma (CHC) is a rare subtype of primary liver cancer (PLC) presenting clinical and pathological distinctions of hepatocellular carcinoma (HCC) and cholangiocarcinoma (CC) simultaneously (1). In 1949, Allen and Lisa (2) categorized CHC as type A (double type), type B (combined type), and type C (mixed type). However, the recent 2019 WHO classification excised other subcategorizations and simplified the definition of CHC as an entity containing an intimate mixture of HCC and intrahepatic cholangiocarcinoma (ICC) components, which was the previously reported Allen C type (3). Due to the changing definitions and nomenclature systems, its reported incidence varied from 0.4% to 14.2% among PLCs (4). It is well known that the most frequent PLCs are HCC and ICC (5).

Extensive studies suggested that CHC tended to occur at chronic liver damage and subsequent cirrhosis, which were the known risk factors of HCC and ICC (6). However, treatment strategies differed substantially among the three diseases. Major hepatic resection with lymph node dissection was the recommended treatment for CHC (7). Trans-arterial chemoembolization (TACE) and locoregional therapies, which can be served as the curative therapy for certain HCCs and ICCs, did not show comparable outcomes in the treatment of CHC (8–11). As for systemic therapy, many chemotherapeutic agents and molecular-targeted drugs for HCC have been developed and drawn encouraging results (sorafenib, nivolumab, etc.) (12, 13). And ICC was reported to harbor the FGFR2 fusions or IDH1/2 mutations, for which infigratinib was developed and now under phase II clinical trial (14). However, systemic therapy regimens for unresectable CHC remain controversial because of its dual nature (15, 16). Liver transplantation had demonstrated inferior survival benefits and higher relapse rates in CHC compared with HCC, which should be avoided in CHC patients so as to allocate liver sources for more appropriate candidates (15, 17). The prognosis of CHC was reported as either intermediate between HCC and ICC or worse than that of both malignancies (18–20). Therefore, imaging differentiation of CHC from HCC and ICC is crucial in terms of proper treatment decision and better survival outcome.

B-mode ultrasound (BMUS) now serves as the first-line imaging modality for tumor detection or follow-up in high-risk patients (21). The development of contrast-enhanced ultrasound (CEUS) enables real-time visualization of the microcirculation within nodules, thus providing more useful diagnostic information. However, owing to the histological diversity and complex existing forms of tumor components, CHC can display either an HCC-like pattern or an ICC-like pattern on CEUS, which makes it difficult to distinguish CHC from HCC and ICC (22). The diagnostic criteria of Liver Imaging Reporting and Data System (LI-RADS) was launched for standardizing the interpretation of liver imaging and classification of hepatic neoplasms (23). However, imaging misclassification has been reported in approximately half of CHC lesions by using the CEUS LI-RADS category (24, 25). The concomitant increase of alpha fetoprotein (AFP) and carbohydrate antigen 19-9 (CA19-9) seems to be of moderate diagnostic value, but the sensitivity (17.8%) was too low (26). Besides, the combination of tumor markers and CEUS findings (i.e., elevated CA19-9 with imaging features of HCC pattern) only reaches moderate success to diagnose CHC (73.3%–76.9% accuracy) (27, 28). Thus, imaging techniques alone or with the help of LI-RADS and tumor markers cannot provide a reliable preoperative diagnosis of CHC and remain inadequate in the instruction of management.

Nomogram is a feasible and relatively objective tool to predict the individual probability of a clinical event, which has been established and validated to be effective in a substantial proportion of cancer types. Here we developed a nomogram based on clinical indicators, BMUS characteristics, and CEUS features that are selected by least absolute shrinkage and selection operator (LASSO) regression analysis, expecting to preoperatively differentiate CHC from HCC and ICC as well as facilitate clinical decision-making.

## Materials and Methods

### Patient Characteristics

**Figure 1:**
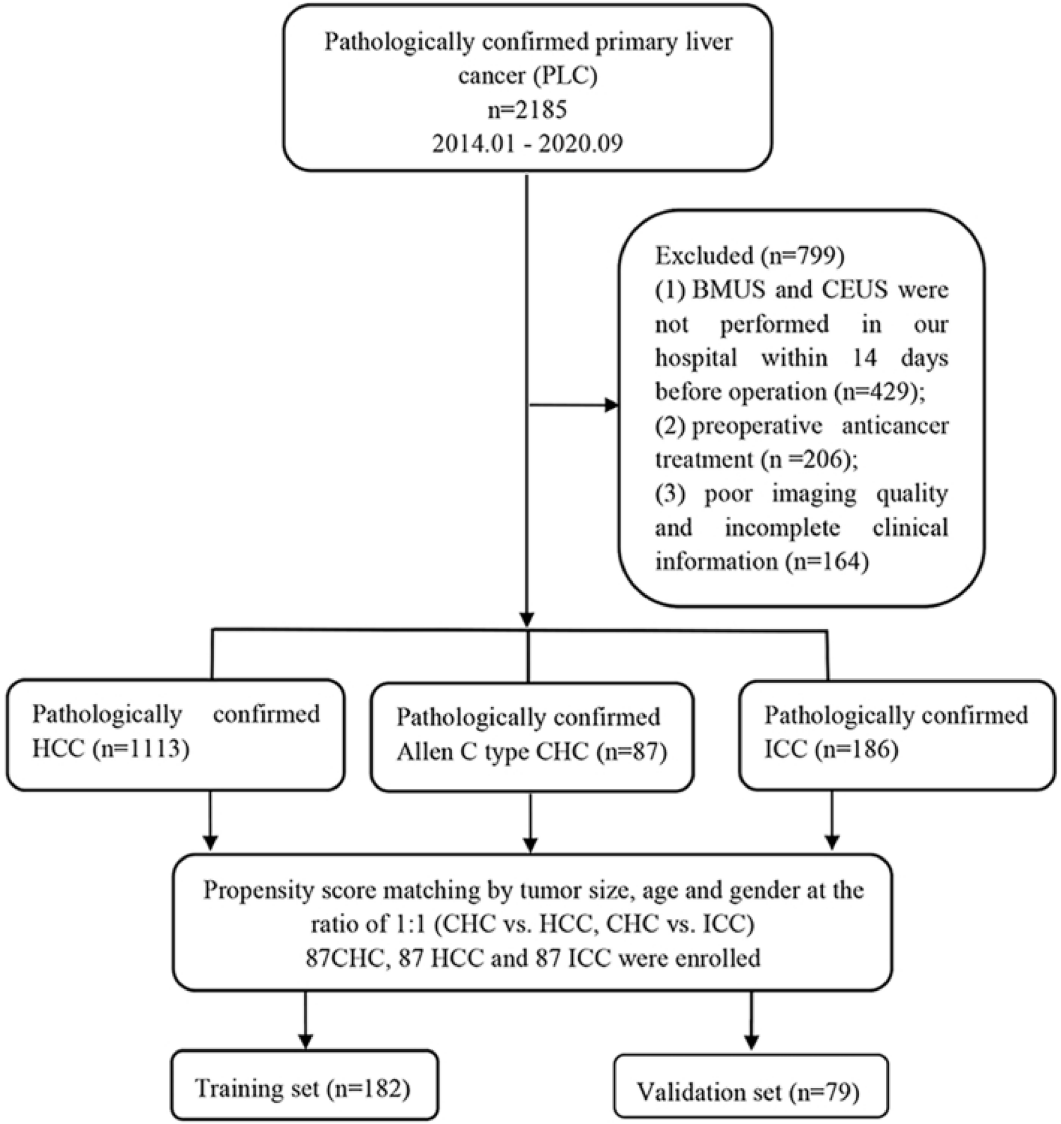
Flowchart of the patient selection process. CHC, combined hepatocellular-cholangiocarcinoma; HCC, hepatocellular carcinoma; ICC, intrahepatic cholangiocarcinoma; BMUS, B-mode ultrasound; CEUS, contrast-enhanced ultrasound.

The implementation of this retrospective study was approved by the ethics committee of our hospital, and informed consent was acquired from the included patients (B2021-082R). Clinical medical data of patients with pathologically confirmed PLCs from January 2014 to September 2020 were reviewed. Inclusion criteria were as follows: 1) the hepatic nodules were confirmed as HCC, ICC, and Allen type C CHC pathologically; 2) BMUS and CEUS were performed within 14 days before operation; 3) without other preoperative anticancer treatment (radiotherapy or systemic chemotherapy, etc.). Exclusion criteria included poor imaging quality and incomplete clinical information. Ultimately, 87 CHCs, 1,113 HCCs, and 186 ICCs met the inclusion criteria. The numbers of patients were then matched using propensity score matching at the ratio of 1:1 by tumor size, age, and gender between CHC and HCC, CHC and ICC, respectively (**Supplementary Table S2**). A total of 261 patients were enrolled in this study and were randomly divided into two datasets: the training cohort (n = 182, 70%) and the validation cohort (n = 79, 30%). The flowchart of patient recruitment is presented in **Figure 1**. Baseline clinical data were obtained from medical records including age, gender, cirrhosis status, hepatitis status, liver function test results, and serum tumor marker levels [AFP, CA19-9, carcinoembryonic antigen (CEA), and des-gamma-carboxyprothrombin (DCP)].

### Ultrasound Imaging Acquisition

BMUS and CEUS examinations were performed by experienced radiologists using a LOGIQ E9 system (GE Healthcare, Milwaukee, WI, USA) equipped with a C1-6 abdominal convex probe and an iU22 system (Philips Bothell, Washington, USA) equipped with a C5-1 convex array transducer. A dose of 1.5~2.4 ml of SonoVue (Bracco, Milan, Italy) was antecubitally injected and immediately followed by a 5.0-ml saline injection. Images were continuously recorded for at least 4~6 min post-injection and stored as digital cine clips.

### Ultrasound Image Assessment

Two radiologists with more than 10-year experience in liver CEUS, both blind the patients’ clinical and pathological information, reviewed the imaging features independently. Disagreement would reach a consensus by discussing with the third radiologist. The CEUS process was classified into three phases: arterial phase (10–20 s~30–45 s), portal venous phase (30–45s~120 s), and delay phases (120 s~4–6 min) (29). Only the nodule with the maximum diameter was enrolled for further analysis for patients with multiple lesions. The following BMUS characteristics of each patient were evaluated and recorded, including the tumor location (right/left/junction of left and right/caudate lobe), number (single or multiple), size, shape (round-like shape was defined as regular, otherwise as irregular), boundary (burr-like, crabfoot-like protrusions or poorly demarcated from the surrounding area were defined as non-smooth margin, otherwise as well-defined boundary), intratumoral echogenicity (hyperechoic, isoechoic, hypoechoic, or heterogeneous echoic), existence of halo sign (defined as a rim of hypoechogenicity surrounded the nodule), presence of intralesion vessels (defined as the existence of blood flow signal detected by color Doppler flow imaging), abnormal lymph nodes (defined as the lymph nodes with a short axis larger than 10 mm), intrahepatic cholangiectasis (defined as the diameter of intrahepatic bile duct greater than 3 mm), and vascular invasion (defined as the visualization of irregular soft tissue in vein).

Concerning the CEUS features, contrast-enhanced intensity of the nodules during the arterial phase, portal venous phase, and delay phase was documented, which was classified as hyperenhancement, isoenhancement, and hypoenhancement after comparing with that of the surrounding parenchyma. The enhanced patterns of tumor were categorized and defined as follows: 1) homogeneous enhancement: the entire nodule exhibits global diffuse enhancement without any perfusion defection; 2) heterogeneous enhancement: the contrast medium distributed inhomogeneously throughout the lesion accompanied by some non-enhanced regions; 3) rim-like enhancement: arterial phase enhancement most pronounced in observation periphery. In addition, the presence of perfusion defection, duration of enhancement (washout time subtracts onset time of enhancement), washout time (within 60 s or not), and presence of marked washout (defined as the lesion appearing as a distinct black defect or presented a “punched-out” appearance within 2 min after contrast injection) were also recorded.

### Histopathologic Analysis

Histopathological examinations were conducted by experienced pathologists in consensus without prior knowledge of the imaging findings and clinical information. Both hematoxylin–eosin staining and immunohistochemical staining (α-fetoprotein, glypican 3, CK7, CK19, etc.) were performed. Finally, the histologic types of lesions were recorded.

### Model Construction and Validation

To decrease the impact of multicollinearity among variables, we performed the LASSO regression to select the most significant predictive features among all the clinical indicators and ultrasonographic characteristics in the training cohort. Multivariate logistic regression analysis was performed to construct a predictive model. The nomogram was then formulated on the basis of the selected variables. Internal and external validations were conducted to determine the diagnostic performance of the predictive model. For internal validation, bootstrap resampling with 1,000 repetitions was performed to avoid overoptimism. The validation set was employed for external validation. Concordance index (C-index) was calculated and receiver operating characteristic (ROC) curves were created to estimate the distinguishability of the nomogram. The C-index ranges from 0.5 to 1. A higher C-index indicates a better predictive power. Calibration of the nomogram for predicting CHC was assessed by the Hosmer–Lemeshow test and the calibration curves. Decision curve analysis (DCA) was carried out to evaluate the clinical usefulness.

### Statistical Analysis

Statistical analysis was performed with SPSS statistical software (version 20.0, IBM, Armonk, NY, USA) and R software (version 4.0.4, R Foundation for Statistical Computing, http://www.r-project.org/, Austria). Seventy percent of patients were assigned to the training dataset and the other 30% were allocated to the validation dataset randomly by R software. Comparison of clinical indicators and BMUS and CEUS imaging features between the CHC and non-CHC groups was executed by using the Mann–Whitney U test, Pearson chi-square test, or Fisher’s exact test. Continuous variables are expressed as median (25th, 75th) and categorical variables as frequency (percentage). *P* values <0.05 suggest statistical significance. Interobserver agreement on the BMUS and CEUS features was measured using kappa (κ) statistics. LASSO regression, nomogram generation, ROC curve analysis, C-index calculation, calibration curve generation, Hosmer–Lemeshow test, and DCA were conducted using R software. The remaining statistical analyses were finished with the help of SPSS statistical software.

The “*glmnet*” function of R was utilized for LASSO regression. The “*glm*” package was taken for univariate and multivariate logistic regression analysis. The “*Hmisc*” package was used for plotting the nomogram. The “*pROC*” function was employed for drawing the ROC curves and calculating the C-index. The “calibration curve” package was used to plot the calibration curves. The “*DecisionCurve*” package was taken for implementing DCA.

## Results

### Patient Characteristics

A total of 261 PLC patients with 261 nodules were enrolled in this study, comprising 87 CHCs, 87 HCCs, and 87 ICCs. Comparisons of clinical characteristics between CHC and non-CHC patients were demonstrated in **Table 1**. Among the recorded clinical information, significant differences were found between the CHC and non-CHC group in age (*P* = 0.028), tumor size (*P* = 0.001), hepatitis B virus (HBV) infection status (*P* = 0.002), cirrhosis status (*P* = 0.001), serum level of AFP ≥20 ng/ml (*P* = 0.001), simultaneous elevation of AFP and CA19-9 (*P* = 0.01), serum level of DCP ≥40 mAU/ml (*P* = 0.014), serum protein electrophoresis (SPE) Alb and α1 level (*P* = 0.037 and *P* > 0.001, respectively). The other clinical indicators did not differ significantly between the two groups. There was no significant difference between the training cohort and the validation cohort with regard to the clinical characteristics (*P* > 0.05) (**Supplementary Table S3**).

**Table 1:** Clinical characteristics of CHC, HCC, and ICC.

Clinical parameters	CHC n = 87	Non-CHC		*P*
		ICC, n = 87	HCC, n = 87	
Tumor size (mm)	27.0 (18.0, 44.0)	41.0 (27.0, 63.0)	30.0 (19.0, 54.0)	<0.01*
Age (years)	58 (48, 65)	63 (53, 69)	58 (50, 66)	0.03*
Gender (male/female)	57/30	49/38	63/24	0.86
Number of nodules (single/multiple)	69/18	75/12	73/14	0.24
Tumor location (right/left/eighter lobe of liver/caudate lobe)	63/23/1/0	54/30/3/0	59/25/3/0	0.43
HBV (+)	82 (94.3)	70 (80.5)	69 (79.3)	<0.01*
HCV (+)	1 (1.1)	0	6 (6.9)	0.50
HEV (+)	8 (9.2)	10 (11.5)	10 (11.5)	0.57
Liver cirrhosis	49 (56.3)	18 (20.7)	43 (49.4)	<0.01*
Tumor marker				
AFP ≥20 (ng/ml)	44 (50.6)	5 (5.7)	47 (54.0)	<0.01*
CA19-9 ≥37 (U/ml)	22 (25.3)	49 (56.3)	10 (11.5)	0.16
CEA ≥5 (ng/ml)	13 (14.9)	21 (24.1)	10 (11.5)	0.56
AFP+CA19-9	11 (12.6)	2 (2.3)	5 (5.7)	0.01*
DCP ≥40 (mAU/ml)	22 (25.3)	6 (6.9)	65 (74.7)	0.01*
Liver functional parameters				
TBL (μmol/L)	12.9 (8.8, 17.1)	11.4 (8.4, 15.0)	13.7 (10.1, 17.5)	0.68
DBL (μmol/L)	4.2 (2.9, 6.4)	3.7 (2.7, 4.6)	4.3 (3.2, 6.2)	0.55
Albumin (g/L)	43.0 (40.0, 47.0)	44.0 (41.0, 47.0)	43.0 (40.0, 47.0)	0.62
Bile acid (μmol/L)	6.5 (4.2, 12.5)	5.1 (3.5, 8.0)	7.2 (4.7, 11.8)	0.34
ALT (U/L)	27.0 (18.0, 42.0)	20.0 (15.0, 26.0)	31.0 (21.0, 47.0)	0.58
AST (U/L)	26.0 (20.0, 33.0)	22.0 (18.0, 29.0)	32.0 (23.0, 45.0)	0.59
AKP (U/L)	78.0 (61.0, 101.0)	85.0 (64.0, 107.0)	87.0 (67.0, 104.0)	0.19
GGT (U/L)	43.0 (26.0, 74.0)	40.0 (25.0, 71.0)	51.0 (28.0, 90.0)	0.82
SPE Alb (%)	59.7 (57.7, 61.3)	58.5 (56.1, 61.0)	59.0 (54.4, 60.9)	0.04*
SPE α1 (%)	3.1 (2.8, 3.4)	3.4 (2.9, 4.2)	3.5 (3.3, 4.0)	<0.01*
SPE α2 (%)	9.3 (8.1, 10.3)	9.5 (8.2, 10.5)	9.4 (8.2, 10.6)	0.38
SPE β (%)	10.5 (9.8, 11.2)	10.6 (9.8, 11.6)	10.3 (9.3, 11.2)	0.92
SPE γ (%)	17.4 (15.3, 19.2)	17.4 (15.6, 19.5)	17.4 (15.7, 21.3)	0.36

Data are presented as median (25th, 75th) and number (percentage); P: statistical difference between CHC and non-CHC. *P < 0.05, significant.

CHC, combined hepatocellular-cholangiocarcinoma; HCC, hepatocellular carcinoma; ICC, intrahepatic cholangiocarcinoma; HBV, hepatitis B virus; HCV, hepatitis C virus; HEV, hepatitis E virus; AFP, alpha fetoprotein; CA19-9, carbohydrate antigen 19-9; CEA, carcinoembryonic antigen; DCP, des-gamma-carboxyprothrombin; TBL, total bilirubin; DBL, direct bilirubin; ALT, alanine aminotransferase; AST, aspartate aminotransferase; AKP, alkaline phosphatase; GGT, γ-glutamyl-transpeptidase; SPE, serum protein electrophoresis.

### B-Mode Ultrasound and Contrast-Enhanced Ultrasound Imaging Characteristics

Substantial or excellent agreement was achieved between the two radiologists, with kappa coefficients ranging from 0.84 to 0.93 (**Supplementary Table S1**). On BMUS, irregular shape and obscure boundary were more frequently observed in the CHC group, whereas acoustic halo sign was more common in non-CHC patients (*P* < 0.05). On CEUS, hyperenhancement in the arterial phase and hypoenhancement in the portal late phase were the primary features of CHC. In the arterial phase, homogeneous hyperenhancement appears in 44.8% (39/87) CHC nodules and 21.3% (37/174) non-CHC nodules. Heterogeneous hyperenhancement was demonstrated in 35.6% (31/87) of CHC lesions in contrast to 67.8% (118/174) of non-CHC lesions. The BMUS and CEUS imaging features of different histo-subtypes of PLC were presented in **Table 2**. No significant difference in the imaging characteristics was found between the two datasets (**Supplementary Table S4**).

**Table 2:** Comparison of qualitative data obtained on BMUS and CEUS features between CHC, HCC, and ICC (%).

BMUS and CEUS features	CHCn = 87	Non-CHC		*P*
		ICC, n = 87	HCC, n = 87	
Echogenicity of nodules (hyper-/iso-/hypo-/mix)	10/8/62/7	11/7/55/14	17/7/57/6	0.57
Irregular shape	53 (60.9)	46 (52.9)	21 (24.1%)	<0.01*
Obscure boundary	72 (82.8)	64 (73.6)	36 (41.4)	<0.01*
Halo sign	27 (31.0)	37 (42.5)	51 (58.6)	<0.01*
Intralesion vessels	48 (55.2)	61 (70.1)	47 (54.0)	0.28
Lymph node metastasis	1 (1.1)	2 (2.3)	0	1.00
Intrahepatic cholangiectasis	6 (6.9)	11 (12.6)	0	0.86
Vascular invasion	4 (4.6)	7 (8.0)	3 (3.4)	0.92
Hyperenhanced in arterial phase	83 (95.4)	78 (89.7)	87 (100.0)	1.00
Hypoenhanced in portal phase	75 (86.2)	76 (87.4)	72 (82.8)	0.80
Hypoenhanced in late phase	80 (92.0)	85 (97.7)	80 (92.0)	0.36
Enhanced pattern				<0.01*
Homogeneous hyperenhancement	39 (44.8)	10 (11.5)	27 (31.0)	
Heterogeneous hyperenhancement	31 (35.6)	61 (70.1)	57 (65.5)	
Rim hyperenhancement	17 (19.5)	16 (18.4)	3 (3.4)	
Duration of enhancement (<30 s)	39 (44.8)	60 (69.0)	21 (24.1)	0.79
Early washout (<60 s)	45 (51.7)	66 (75.9)	30 (34.5)	0.60
Marked washout	39 (44.8)	64 (73.6)	10 (11.5)	0.72
Perfusion defect	23 (26.4)	41 (47.1)	23 (26.4)	0.10

Data are presented as number (percentage); P: Statistical difference between CHC and non-CHC. *P < 0.05, significant.

CHC, combined hepatocellular-cholangiocarcinoma; HCC, hepatocellular carcinoma; ICC, intrahepatic cholangiocarcinoma.

### Prediction Model and Nomogram Construction and Validation

**Figure 2:**
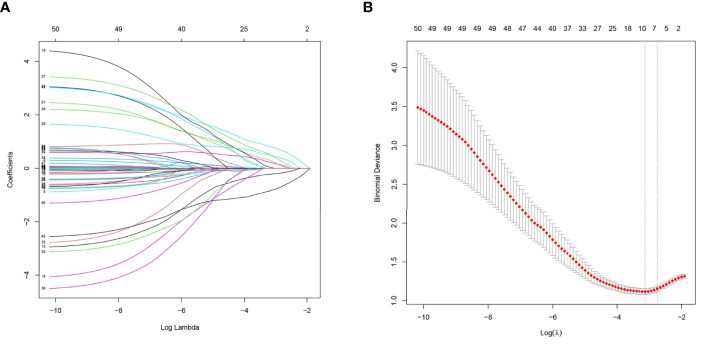
**(A)** Ultrasound imaging features and clinical characteristics selection using the least absolute shrinkage and selection operator (LASSO) logistic regression model in the training cohort. **(B)** A 10-fold cross-validation method was used in this model to minimize the binomial deviation by adjusting different parameters of λ so as to find out predictors with higher diagnostic value. Seven features with non-zero coefficients were eventually selected at the optimal λ.

**Figure 3:**
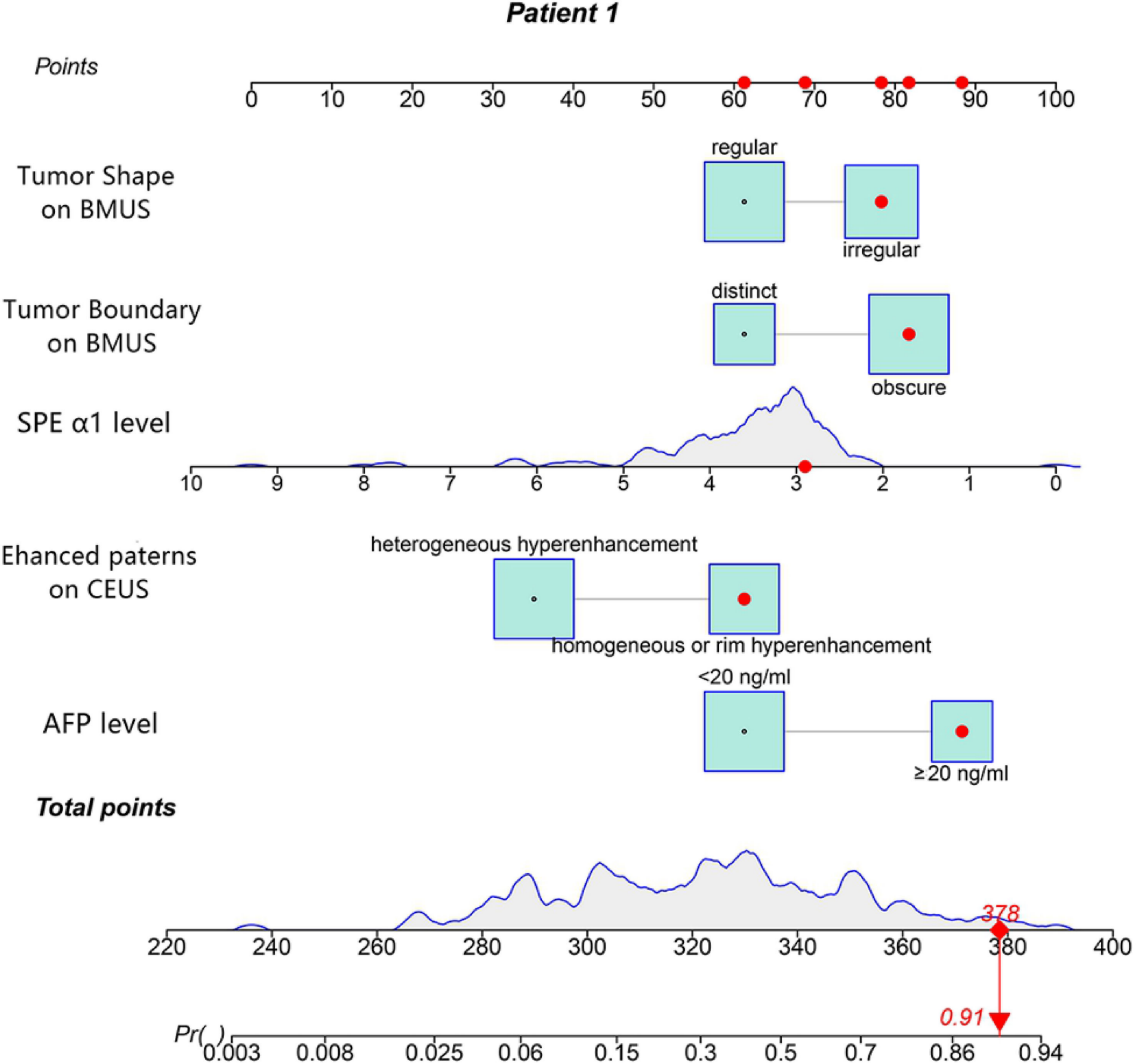
The nomogram was developed in the training cohort. Values for each predictor are located on each variable axis, which correspond to a point at the top of the graph. Points for all variables are added and translated into the possibility of a combined hepatocellular-cholangiocarcinoma (CHC) diagnosis.

**Figure 4:**
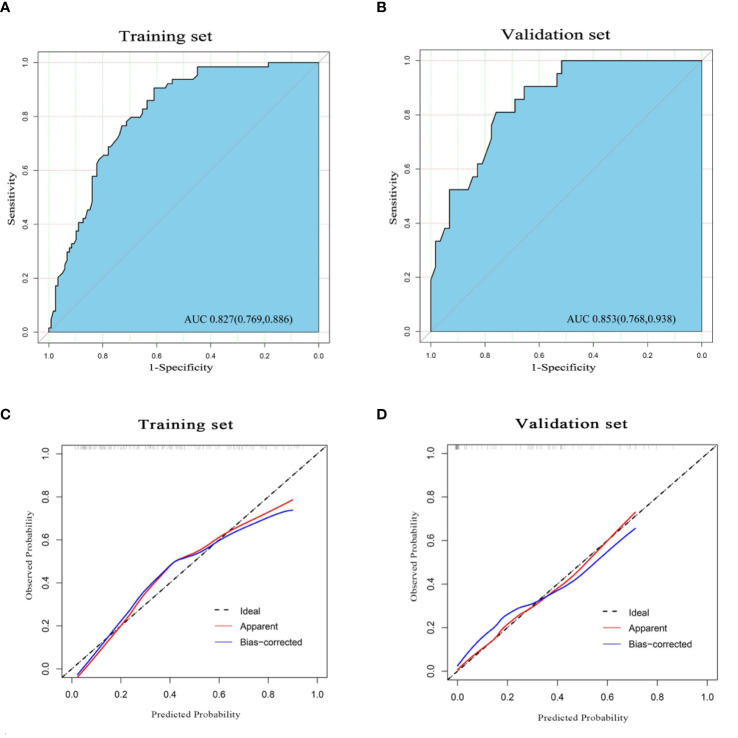
The receiver operating characteristic (ROC) curve of the nomogram in the training set **(A)** and validation set **(B)**, with the respective area under the curve (AUC) of 0.827 and 0.853, indicating a high diagnostic value of the nomogram. The calibration curve of the nomogram in the training cohort **(C)** and validation cohort **(D)**. The apparent line (red) and the bias-corrected line (blue) in the calibration curves ideally matched with the actual line (dash line), which indicates good consistency between the prediction and actual observation in both datasets.

**Figure 5:**
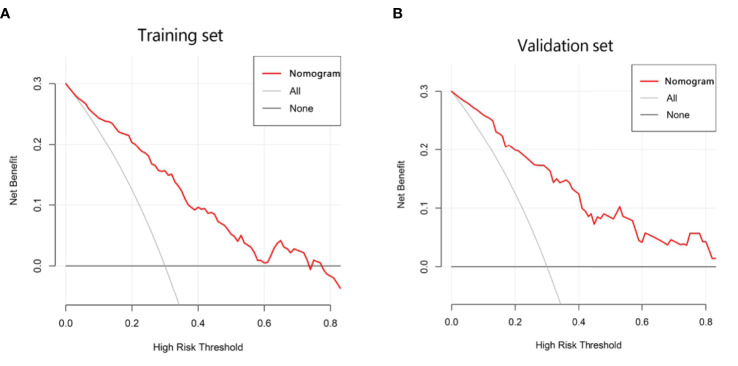
Decision curve analysis (DCA) of the nomogram in the training **(A)** and validation cohorts **(B)**, which visually indicated that the nomogram conferred high clinical net benefit compared to the treat-all-patients strategy (solid gray line) or treat-none strategy (horizontal solid black line).

The predictors strongly associated with the possibility of CHC diagnosis were identified by LASSO regression in the training set, including clinical indicators of elevated AFP level, SPE α1 level, HBV infection and liver cirrhosis, BMUS features of irregular shape and obscure boundary, and the enhanced pattern on CEUS (**Figures 2A, B** **)**. According to the multivariate logistic regression analysis, HBV infection and liver cirrhosis were not the independent predictors for the diagnosis of CHC (*P* > 0.05) (**Table 3**). The remaining variables were then incorporated into the predictive model, and a nomogram was generated (**Figure 3**). The nomogram reflected a high overall classification performance for differentiating CHC from non-CHC nodules, with the C-index of 0.8275 (95% CI: 0.7687–0.8862) in the training cohort and 0.8530 (95% CI: 0.7677–0.9383) in the validation cohort after bootstrapping with 1,000 replications. This was also confirmed by the ROC curves (**Figures 4A, B** **)**. The calibration curves ideally matched with the identity line (45° line) (**Figures 4C, D** **)**, which indicates an excellent fit between the prediction and actual observation in both datasets. The Hosmer–Lemeshow χ^2^ in the training and validation sets was 8.7968 (*P* = 0.359) and 3.0907 (*P* = 0.9285), respectively, which also confirmed that a well-fitting model has been obtained. In addition, the decision curve (**Figures 5A, B** **)** in both the training and validation cohorts showed that the nomogram possessed a high net benefit compared to the treat-all-patients strategy or treat-none strategy at different threshold probabilities.

**Table 3:** Results of LASSO regression analysis in the training cohort.

Parameter	OR	95% CI	*P*
AFP ≥20 (ng/ml)	3.71	1.72, 8.28	<0.01*
SPE α1	0.62	0.38, 0.93	0.03*
HBV (+)	3.08	0.9, 12.25	0.087
Liver cirrhosis	1.73	0.78, 3.82	0.17
Irregular shape	3.25	1.41, 7.90	<0.01*
Obscure boundary	2.55	1.07, 6.38	0.04*
Heterogeneous hyperenhancement	0.22	0.10, 0.47	<0.01*

*P < 0.05, significant.

OR, odds ratio; CI, confidence interval; AFP, alpha fetoprotein; SPE, serum protein electrophoresis; HBV, hepatitis B virus; LASSO, least absolute shrinkage and selection operator.

## Discussion

CHC is the third most common PLC with the incidence only second to HCC and ICC. Preoperative identification of CHC and non-CHC PLC on imaging is necessary with regard to the proper treatment decision for better prognosis. However, as a biphasic tumor formed by a mixture of hepatocellular and biliary components, the imaging findings of CHC have always been elusive.

A nomogram is able to quantify the risk of a clinical event through an intuitive graph of a statistical predictive model, which has been widely developed for the auxiliary diagnosis and prognostic prediction of malignancy (30). Wang et al. (31) established a nomogram based on clinical indicators to differentiate CHC from ICC, which achieved a good discriminating capability (C-index of 0.796). To make preoperative identification of CHC and fully exploit the advantage of BMUS and CEUS, we constructed a nomogram incorporating clinical indexes along with imaging characteristics of BMUS and CEUS. The nomogram integrated five parameters selected by LASSO regression, including irregular shape and obscure boundary on BMUS, the enhanced pattern on CEUS, and the serologic test of elevated AFP level as well as SPE α1 level. The utilization of LASSO regression effectively reduced the impact of multicollinearity and model overfitting (32).

Malignancy is characterized by the presence of irregular shape and ill-defined boundary. Ye et al. (33) found that a higher proportion of ill-defined boundaries was observed in CHCs than that in HCCs. In accordance with their study, indistinct margin and irregular shape presented more frequently in the CHC and ICC lesions than in the HCC nodules. One plausible reason might be that cholangiocarcinoma-containing tumors, whose tumor cell partly arises from cholangiocyte, have the property of infiltrative progression (34). In the present study, homogeneous hyperenhancement was more frequently found in CHC, whereas heterogeneous hyperenhancement was more commonly identified in HCC and ICC. Inconsistently, the studies from Huang et al. (28) and Zhang et al. (26) found that heterogeneous hyperenhancement was more common in CHC and ICC, and homogeneous hyperenhancement was more frequently observed in HCC, which might be due to the high degree of heterogeneity in CHC. It was believed that the CEUS patterns of CHC nodules might be influenced by the relative proportions of the HCC/ICC components within nodules (35). Regardless, the different CEUS patterns of nodules were verified as a predictor in distinguishing different subtypes of PLC, the mechanism of which needs be further investigated. AFP is the widely used tumor marker for PLC, especially for the HCC-containing tumors (36). In our study, the elevation of AFP was more common in the HCC and CHC than in ICC, which was consistent with the study by Zhang et al. (26). They found that elevated AFP was observed in 71.1% of HCC, 55.6% of CHC, and 2.2% of ICC. Reason for such a discrepancy might be that AFP was synthesized by liver cells, whereas ICC mainly originated from the epithelial cells of the intrahepatic bile duct. This finding may help rule out the ICC diagnosis among PLCs. SPE is an inexpensive and useful tool to assess liver function. An increase of the α1 globulins and α2 globulins band was observed in many malignant tumors due to the increase of acute-phase proteins (37). Differences of SPE Alb and SPE α1 level between the CHC group and non-CHC group were observed in our study, and SPE α1was further confirmed as a predictor by LASSO regression to identify CHC and non-CHC lesions. However, the cause of this discrepancy has not yet been determined.

**Figure 6:**
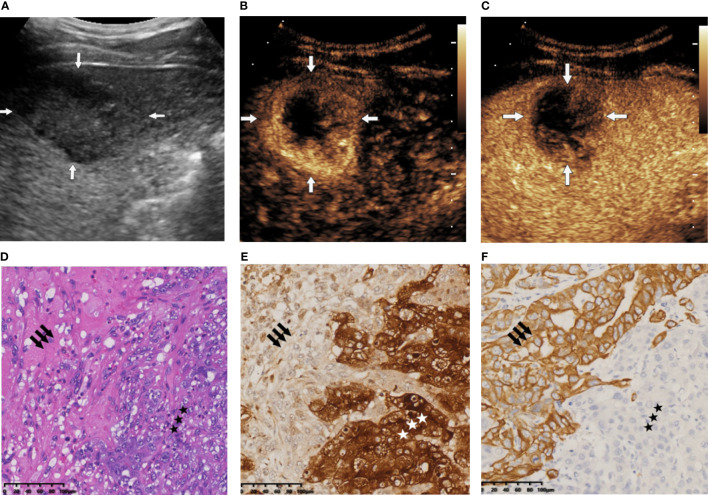
**(A)** B-mode ultrasound (BMUS) examination of patient 1. A mass in the right hepatic lobe with an oval shape and an obscure boundary was observed. **(B)** The tumor displayed rim hyperenhancement in the arterial phase on contrast-enhanced ultrasound (CEUS). **(C)** The lesion exhibited early (washout onset time, 33 s) and marked washout (arrow) during the portal venous phase. **(D)** Histopathological examination confirmed the diagnosis of combined hepatocellular cholangiocarcinoma (CHC), with coexistence of hepatocytic (star) and cholangiocytic (arrow) components (H&E staining; magnification, ×20). **(E)** Immunohistochemical staining indicated that the hepatocytic elements were positive for glypican 3 (magnification, ×20). **(F)** The cholangiocytic component was positive for CK19 (magnification, ×20).

The nomogram we established is an intuitive and easy-to-use tool for the individualized prediction of a CHC diagnosis. It performed well in differentiating CHC from non-CHC nodules, achieving the C-index of 0.8275 in the training set and 0.8530 in the validation set. Good agreement between predictions and observations underlined the reliability and repeatability of this nomogram. The nomogram will help identify the CHC nodules previously misdiagnosed as HCC or ICC and lead to more appropriate treatment plans. For instance, a patient with elevated AFP level (25.4 ng/ml) and SPE α1 level of 2.9% revealed a 45 × 30-mm mass on BMUS with irregular shape and obscure boundary (**Figure 6A**). The nodule displayed rim hyperenhancement in the arterial phase on CEUS (**Figures 6B, C** **)**. On the basis of the nomogram, the possibility of CHC diagnosis was 0.91 (**Figure 3**). Postoperative pathological examination confirmed a diagnosis of CHC (**Figures 6D–F**). Therefore, curative hepatectomy with lymph node dissection would be the best treatment option for this patient. DCA also found that this CEUS nomogram added more benefit than either the treat-none scheme or the treat-all-patients strategy. Beyond that, the ability of the nomogram in evaluating the personalized probability of CHC diagnosis will facilitate patient selection for prospective clinical trials.

Previously, researchers have attempted to identify the three different types of PLCs by imaging modalities. Wang et al. (38) tried to identify HCC, ICC, and CHC based on their preoperative CT and MRI, through which they found that imaging features of capsular retraction, biliary dilatation, pseudocapsule, rim enhancement, and abnormal perfusion differed significantly between the three malignancies, and CHC was intermediate between HCC and ICC. However, they also pointed out that the enhancement pattern of CHC resembled HCC in most cases. Immediately after, LI-RADS was brought into the attempts of CHC diagnosis. Choi et al. (39) evaluated the images of 194 PLC nodules (53 with CHC, 44 with ICC, and 97 with HCC) with gadoxetic acid-enhanced MRI and classified them according to the LI-RADS; they found that the misdiagnosis of HCC was mainly due to the confusing imaging manifestation of CHC, since 85% of false-positive diagnosis of HCC (LR-5) was CHC. Compared with CT or MRI, ultrasound technology features simple operation and real-time observation, which played an important role in the diagnosis and intervention of liver neoplasms.

The ability of CEUS in making differentiation of CHC from HCC and ICC was also investigated. The study of Sagrini et al. (40) compared the ability of different imaging modalities (CEUS, CT, and MRI) in making diagnosis of CHC, which revealed that CEUS possessed strong diagnostic ability in distinguishing malignant from benign liver lesions while it failed to make a definitive diagnosis of CHC in most cases. On this basis, a combination of CEUS and tumor markers was carried out (simultaneous elevation of AFP and CA19-9 or elevated tumor markers in discordance with the imaging findings). However, this criterion also presented limited diagnostic capacity, with the accuracy of 71.1%–74.4% (22, 26, 28). Recent studies further investigated the value of CEUS LI-RADS combined with tumor markers in making a diagnosis of CHC. Nevertheless, the accuracy (76.9%) showed no significant improvement compared with CEUS only (27). Comparing with the mentioned diagnostic modalities, our nomogram not only incorporated tumor markers and CEUS characteristics but also contained BMUS features and indicator of liver function test, which were the underestimated factors in previous studies, and the diagnostic power was therefore improved.

Ultrasound radiomics was the major research focus currently, which greatly enhanced the imaging diagnostic efficiency for hepatic tumors. Peng et al. (41) extracted radiomics features from the grayscale ultrasound images of 668 PLCs and constructed the radiomics model. Their model displayed a high diagnostic value in identifying HCC, ICC, and CHC, with AUCs of 0.920 (training cohort) and 0.728 (test cohort). Nevertheless, the application of the radiomics models required specific techniques and software, which was not user-friendly (42). On the contrary, our nomogram not only shows high diagnostic effectiveness but also incorporates few predictors, which will simplify the diagnostic process and be more applicable for less experienced physicians. Additionally, all the three imaging features and two clinical indicators are convenient to obtain from preoperative examination, making the nomogram with high clinical practicability.

Despite the good performance, certain limitations existed in the present study. First, as a single-center study, additional multicenter prospective studies are necessary so as to validate the diagnostic ability of the nomogram. Second, the evaluation of imaging characteristics showed operator dependence. Subjectivity cannot be eliminated entirely. Lastly, heterogeneity of the ultrasound images was inevitable in this retrospective study, since the ultrasound examinations were conducted in different machines by different radiologists. Therefore, a prospective study with designated radiologists and specific ultrasound machine is mandatory.

## Conclusions

In conclusion, we developed and validated a nomogram based on ultrasonographic features and clinical characteristics to differentiate CHC from HCC and ICC. Our results suggested that the nomogram had promising predictive power and high clinical practicability, which will be helpful in making preoperative diagnosis and selecting appropriate treatments.

## Data Availability Statement

The original contributions presented in the study are included in the article/**Supplementary Material**. Further inquiries can be directed to the corresponding author.

## Ethics Statement

The studies involving human participants were reviewed and approved by the ethics committee of Zhongshan Hospital, Fudan University. The patients/participants provided their written informed consent to participate in this study. Written informed consent was obtained from the individual(s) for the publication of any potentially identifiable images or data included in this article.

## Author Contributions

YC made contribution to the study designed, statistical analysis, and article drafting. QL, WZ, YD, JC, and WW contributed to the ultrasound image acquirement. QL and WW critically reviewed the article and made revisions. All authors participated sufficiently in the study and gave approval of the submitted version.

## Funding

The study was supported by the National Natural Science Foundation of China (Grant no. 82071924); Natural Science Foundation Project of Shanghai (Grant no. 20ZR1452800); Clinical Research Plan of Shanghai Hospital Development Center (Grant no. SHDC2020CR1031B), and Shanghai Municipal Key Clinical Specialty (Grant no. shslczdzk03501).

## Conflict of Interest

The authors declare that the research was conducted in the absence of any commercial or financial relationships that could be construed as a potential conflict of interest.

## Publisher’s Note

All claims expressed in this article are solely those of the authors and do not necessarily represent those of their affiliated organizations, or those of the publisher, the editors and the reviewers. Any product that may be evaluated in this article, or claim that may be made by its manufacturer, is not guaranteed or endorsed by the publisher.
