# Supplementary material for: Preoperative Differentiation of Combined Hepatocellular-Cholangiocarcinoma From Hepatocellular Carcinoma and Intrahepatic Cholangiocarcinoma: A Nomogram Based on Ultrasonographic Features and Clinical Indicators
Source: Front Oncol. 2022 Feb 15;12:757774. doi: 10.3389/fonc.2022.757774 (PMC8885729; doi:10.3389/fonc.2022.757774)
Supplement: Supplementary file 1 [file DataSheet_1.zip › Supplementary Table 1.DOCX]

Supplementary Table 1 Kappa value of the Pre-contrast and contrast-enhanced ultrasound imaging features assessment between two radiologists

| Parameter | kappa value |
| --- | --- |
| Echogenicity of nodules(hyper-/iso-/hypo-/Mix) | 0.93 |
| Irregular shape | 0.87 |
| Obscure boundary | 0.89 |
| Halo sign | 0.90 |
| Intra-lesion vessels | 0.90 |
| Lymph node metastasis | 0.88 |
| Intrahepatic cholangiectasis | 0.88 |
| Vascular invasion | 0.86 |
| Hyper-enhanced in arterial phase | 0.92 |
| Hypo-enhanced in portal phase | 0.86 |
| Hypo-enhanced in late phase | 0.84 |
| Enhanced pattern | 0.85 |
| Duration of enhancement (< 30s) | 0.87 |
| Early washout(<60s) | 0.87 |
| Marked washout | 0.85 |
| Perfusion defect | 0.91 |

Slight agreement: 0 < kappa (κ) < 0.20; Fair agreement: 0.20 < κ < 0.40; Moderate agreement: 0.40 < κ < 0.60; Substantial agreement: 0.60 < κ < 0.80; Perfect agreement: 0.80 < κ < 1
