# Supplementary material for: Preoperative Differentiation of Combined Hepatocellular-Cholangiocarcinoma From Hepatocellular Carcinoma and Intrahepatic Cholangiocarcinoma: A Nomogram Based on Ultrasonographic Features and Clinical Indicators
Source: Front Oncol. 2022 Feb 15;12:757774. doi: 10.3389/fonc.2022.757774 (PMC8885729; doi:10.3389/fonc.2022.757774)
Supplement: Supplementary file 1 [file DataSheet_1.zip › Supplementary Table 2.DOCX]

Supplement Table 2 Base line Characteristics of Patients before and after Propensity Score Analysis

| Clinical parameters | Before propensity score matching | | | P value | After propensity score matching | | | P value |
| --- | --- | --- | --- | --- | --- | --- | --- | --- |
|  | CHC, n = 87 | Non-CHC | |  | CHC, n = 87 | Non-CHC | |  |
|  |  | ICC, n = 186 | HCC, n = 1113 |  |  | ICC, n = 87 | HCC, n = 87 |  |
| Tumor size(cm) | 27.0(18.0,44.0) | 36.5(22.8,60.0) | 37.0(23.0,57.0) | <0.01* | 27.0(18.0,44.0) | 41.0(27.0,63.0) | 30.0(19.0,54.0) | <0.01* |
| Age(years) | 58(48,65) | 62(52,68) | 56(47,65) | 0.76 | 58(48,65) | 63(53,69) | 58(50,66) | 0.03* |
| Gender(male/female) | 57/30 | 129/57 | 768/345 | 0.49 | 57/30 | 49/38 | 63/24 | 0.86 |
| Number of nodules  (single/multiple) | 69/18 | 156/30 | 928/185 | 0.32 | 69/18 | 75/12 | 73/14 | 0.24 |
| HBV (+) | 82(94.3) | 134(72.0) | 980(88.1) | 0.03 | 82(94.3) | 70(80.5) | 69(79.3) | <0.01* |
| HCV (+) | 1(1.1) | 4(2.2) | 30(2.7) | 0.62 | 1(1.1) | 0 | 6(6.9) | 0.50 |
| HEV (+) | 8(9.2) | 18(9.7) | 80(7.2) | 0.58 | 8(9.2) | 10(11.5) | 10(11.5) | 0.57 |
| Liver cirrhosis | 49(56.3) | 71(38.2) | 655(58.8) | 0.94 | 49(56.3) | 18(20.7) | 43(49.4) | <0.01* |
| Tumor marker |  |  |  |  |  |  |  |  |
| AFP≥20 (ng/ml) | 44(50.6) | 38(20.4) | 569(51.1) | 0.49 | 44(50.6) | 5(5.7) | 47(54.0) | <0.01* |
| CA199≥37(U/ml) | 22(25.3) | 81(43.5) | 165(14.8) | 0.15 | 22(25.3) | 49(56.3) | 10(11.5) | 0.16 |
| CEA≥5 (ng/ml) | 13(14.9) | 45(24.2) | 153(13.7) | 0.94 | 13(14.9) | 21(24.1) | 10(11.5) | 0.56 |
| AFP+CA199 | 11(12.6) | 13(7.0) | 74(6.6) | 0.04 | 11(12.6) | 2(2.3) | 5(5.7) | 0.01* |

Data are presented as median (25th, 75th) and number (percentage); P: statistical difference between CHC and non-CHC. *P ˂ 0.05, significant; CHC: combined hepatocellular-cholangiocarcinoma; HCC: hepatocellular carcinoma; ICC: intrahepatic cholangiocarcinoma; HBV: hepatitis B virus; HCV: hepatitis C virus; HEV: hepatitis E virus; AFP: alpha fetoprotein; CA199: carbohydrate antigen 19-9; CEA: carcinoembryonic antigen
