# Supplementary material for: Preoperative Differentiation of Combined Hepatocellular-Cholangiocarcinoma From Hepatocellular Carcinoma and Intrahepatic Cholangiocarcinoma: A Nomogram Based on Ultrasonographic Features and Clinical Indicators
Source: Front Oncol. 2022 Feb 15;12:757774. doi: 10.3389/fonc.2022.757774 (PMC8885729; doi:10.3389/fonc.2022.757774)
Supplement: Supplementary file 1 [file DataSheet_1.zip › Supplementary Table 3.DOCX]

Supplement Table 3 Clinical characteristics of CHC, HCC and ICC in the training set and validation set

| Clinical parameters | Training set | | | Validation set | | | *P* |
| --- | --- | --- | --- | --- | --- | --- | --- |
|  | CHC  n = 66 | Non-CHC | | CHC  n = 21 | Non-CHC | |  |
|  |  | ICC, n = 65 | HCC, n = 51 |  | ICC, n = 22 | HCC, n = 36 |  |
| Tumor size(cm) | 26.0(16.0,44.3) | 40.0(27.0,57.5) | 33.0(21.0,54.0) | 30 (19.0,43.5) | 45.5(27.0,64.0) | 29.5(17.0,55.5) | 0.63 |
| Age(years) | 55(48,65) | 64(54,69) | 56(50,68) | 60(51,65) | 63(53,70) | 58(50,65) | 0.82 |
| Gender(male/female) | 43/23 | 37/28 | 40/11 | 14/7 | 12/10 | 23/13 | 0.54 |
| Number of nodules  (single/multiple) | 54/12 | 55/10 | 45/6 | 15/6 | 20/2 | 28/8 | 0.33 |
| Tumor location  (right/left/eighter lobe of liver) | 51/14/1 | 43/20/2 | 35/14/2 | 12/9/0 | 11/10/1 | 24/11/1 | 0.16 |
| HBV (+) | 61(92.4) | 51(78.5) | 39(76.5) | 21(100.0) | 19(86.4) | 30(83.3) | 0.25 |
| HCV (+) | 1(1.5) | 0 | 3(5.9) | 0 | 0 | 3(8.3) | 0.75 |
| HEV (+) | 4(6.1) | 8(12.3) | 6(11.8) | 4(19.0) | 2(9.1) | 4(11.1) | 0.51 |
| Liver cirrhosis | 36(54.5) | 13(20.0) | 25(49.0) | 13(61.9) | 5(22.7) | 18(50.0) | 0.46 |
| Tumor marker |  |  |  |  |  |  |  |
| AFP ≥20 (ng/ml) | 37(56.1) | 5(7.7) | 25(49.0) | 7(33.3) | 0 | 22(61.1) | 0.99 |
| CA19-9≥37(U/ml) | 17(25.8) | 37(56.9) | 7(13.7) | 5(23.8) | 12(54.5) | 3(8.3) | 0.19 |
| CEA≥5 (ng/ml) | 11(16.7) | 17(26.2) | 5(9.8) | 2(9.5) | 4(18.2) | 5(13.9) | 0.40 |
| AFP+CA199 | 11(16.7) | 2(3.1) | 3(5.9) | 0 | 0 | 2(5.6) | 0.12 |
| DCP≥40 (mAU/ml) | 19(28.8) | 3(4.6) | 41(80.4) | 3(14.3) | 3(13.6) | 24(66.7) | 0.60 |
| Liver functional parameters |  |  |  |  |  |  | 0.46 |
| TBL (μmol/L) | 13.0(9.1,16.8) | 11.8(8.5,15.1) | 13.3(10.1,16.5) | 10.5(7.6,18.0) | 10.7(8.3,13.8) | 14.0(10.0,18.2) | 0.84 |
| DBL(μmol/L) | 4.3(3.0,6.1) | 3.8(3.0,4.7) | 4.2(3.2,6.3) | 3.8(2.5,7.4) | 3.4(2.5,4.4) | 4.3(3.2,6.2) | 0.82 |
| Albumin (g/L) | 43.5(41.0,47.0) | 44.0(41.0,47.0) | 42.0(40.0,45.0) | 41.0(39.0,46.0) | 43.5(41.8,47.0) | 44.0(39.0,48.0) | 0.50 |
| Bile acid (μmol/L) | 6.5(3.8,12.5) | 5.1(3.3,7.8) | 7.2(4.9,12.6) | 6.5(4.8,18.8) | 5.5(3.9,8.2) | 7.3(4.4,11.0) | 0.57 |
| ALT (U/L) | 28.0(18.8,42.3) | 20.0(15.0,27.5) | 32.0(22.0,51.0) | 21.0(16.0,42.0) | 18.5(13.8,25.3) | 26.5(20.0,46.8) | 0.77 |
| AST (U/L) | 26.5(20.0,33.0) | 23.0(17.0,28.5) | 31.0(22.0,41.0) | 25.0(20.5,32.5) | 21.0(18.0,32.3) | 34.5(25.5,54.8) | 0.12 |
| AKP(U/L) | 77.0(60.8,94.5) | 86.0(65.5,108.0) | 87.0(67.0,106.0) | 92.0(66.5,128.5) | 76.0(67.8,105.5) | 86.5(67.3,102.5) | 0.50 |
| GGT(U/L) | 42.5(26.0,67.8) | 40.0(25.0,75.5) | 51.0(33.0,80.0) | 67.0(32.0,82.5) | 40.5(24.0,75.3) | 52.0(22.5,105.5) | 0.44 |
| SPE Alb (%) | 59.7((57.5,61.2) | 58.5(56.0,60.4) | 59.3(54.8,61.8) | 59.7(57.3,62.2) | 58.8(56.5,61.6) | 58.4(54.2,60.9) | 0.68 |
| SPR α1(%) | 3.1(2.8,3.6) | 3.4(2.9,4.2) | 3.6(3.3,4.1) | 3.1(2.8,3.4) | 3.4(3.0,3.8) | 3.4(3.3,4.0) | 0.80 |
| SPE α2(%) | 8.9(8.1,10.7) | 9.5(8.1,10.5) | 9.3(8.2,10.5) | 9.2(7.8,10.0) | 9.9(8.5,11.2) | 9.4(8.5,10.6) | 0.78 |
| SPE β (%) | 10.5(9.9,11.2) | 10.7(9.8,11.7) | 10.3(9.5,11.3) | 10.1(9.2,10.9) | 10.4(9.9,10.9) | 10.1(9.2,11.2) | 0.07 |
| SPE γ (%) | 17.3(15.1,19.2) | 17.5(15.9,19.6) | 17.1(15.2,20.6) | 17.3(15.7,19.2) | 16.2(15.4,19.1) | 18.2(16.3,22.9) | 0.41 |

Data are presented as median (25th, 75th) and number (percentage); P: Statistical difference between the training set and validation set. *P ˂ 0.05, significant; CHC: combined hepatocellular-cholangiocarcinoma; HCC: hepatocellular carcinoma; ICC: intrahepatic cholangiocarcinoma; HBV: hepatitis B virus; HCV: hepatitis C virus; HEV: hepatitis E virus; AFP: alpha fetoprotein; CA199: carbohydrate antigen 19-9; CEA: carcinoembryonic antigen; DCP: des-gamma-carboxyprothrombin; TBL: total bilirubin; DBL: Direct bilirubin; ALT: alanine aminotransferase; AST: aspartate aminotransferase; AKP: alkaline phosphatase; GGT: γ-glutamyl-transpeptidase; SPE: serum protein electrophoresis
