# Supplementary material for: Preoperative Differentiation of Combined Hepatocellular-Cholangiocarcinoma From Hepatocellular Carcinoma and Intrahepatic Cholangiocarcinoma: A Nomogram Based on Ultrasonographic Features and Clinical Indicators
Source: Front Oncol. 2022 Feb 15;12:757774. doi: 10.3389/fonc.2022.757774 (PMC8885729; doi:10.3389/fonc.2022.757774)
Supplement: Supplementary file 1 [file DataSheet_1.zip › Supplementary Table 4.DOCX]

Supplement Table 4 Comparison of qualitative data obtained on BMUS and CEUS features between CHC, HCC and ICC

| Clinical parameters | Training set | | | Validation set | | | *P* |
| --- | --- | --- | --- | --- | --- | --- | --- |
|  | CHC  n = 66 | Non-CHC | | CHC  n = 21 | Non-CHC | |  |
|  |  | ICC, n = 65 | HCC, n = 51 |  | ICC, n = 22 | HCC, n = 36 |  |
| Echogenicity of nodules(hyper-/iso-/hypo-/Mix) | 7/7/48/4 | 6/6/43/10 | 9/4/33/5 | 3/1/14/3 | 5/1/12/4 | 8/3/24/1 | 0.35 |
| Irregular shape | 38(57.6) | 36(55.4) | 9(17.6) | 15(71.4) | 10(45.5) | 12(33.3) | 0.86 |
| Obscure boundary of nodules | 51(77.3) | 46(70.8) | 18(35.3) | 21(100.0) | 18(81.8) | 18(50.0) | 0.16 |
| Halo sign | 23(34.8) | 27(41.5) | 32(62.7) | 4(19.0) | 10(45.5) | 19(52.8) | 0.62 |
| Intra-lesion vessels | 35(53.0) | 45(69.2) | 30(58.8) | 13(61.9) | 16(72.7) | 17(47.2) | 0.74 |
| Lymph node metastasis | 1(1.5) | 2(3.1) | 0 | 0 | 0 | 0 | 0.56 |
| Intrahepatic cholangiectasis | 4(6.1) | 9(13.8) | 0 | 2(9.5) | 2(9.1) | 0 | 0.72 |
| Vascular invasion | 3(4.5) | 5(7.7) | 1(2.0) | 1(4.8) | 2(9.1) | 2(5.6) | 0.65 |
| Hyper-enhanced in arterial phase | 63(95.5) | 59(90.8) | 51(100.0) | 20(95.2) | 19(86.4) | 36(100.0) | 1.00 |
| Hypo-enhanced in portal phase | 56(84.8) | 57(87.7) | 44(86.3) | 19(90.5) | 19(86.4) | 28(77.8) | 0.57 |
| Hypo-enhanced in late phase | 61(92.4) | 63(96.9) | 47(92.2) | 19(90.5) | 22(100.0) | 33(91.7) | 0.93 |
| Enhanced pattern |  |  |  |  |  |  | 0.5 |
| Homogeneous hyperenhancement | 29(43.9) | 8(12.3) | 14(27.5) | 10(47.6) | 2(9.1) | 13(36.1) |  |
| Heterogeneous hyperenhancement | 24(36.4) | 44(67.7) | 35(68.6) | 7(33.3) | 17(77.3) | 22(61.1) |  |
| Rim hyperenhancement | 13(19.7) | 24(36.4) | 2(3.9) | 4(19.0) | 3(13.6) | 1(2.8) |  |
| Duration of enhancement (< 30s) | 31(47.0) | 47(72.3) | 16(31.4) | 8(38.1) | 13(59.1) | 5(13.9) | 0.01* |
| Early washout (<60s) | 34(51.5) | 51(78.5) | 23(45.1) | 11(52.4) | 15(68.2) | 7(19.4) | 0.01* |
| Marked washout | 31(47.0) | 47(72.3) | 6(11.8) | 8(38.1) | 17(77.3) | 4(11.1) | 0.16 |
| Perfusion defect | 18(27.3) | 32(49.2) | 13(25.5) | 5(23.8) | 9(40.9) | 10(27.8) | 0.51 |

Data are presented as number (percentage); P: Statistical difference between the training set and validation set. *P ˂ 0.05, significant; CHC: combined hepatocellular- cholangiocarcinoma; HCC: hepatocellular carcinoma; ICC: intrahepatic cholangiocarcinoma
